# A deep learning-based approach to diagnose mild traumatic brain injury using audio classification

**DOI:** 10.1371/journal.pone.0274395

**Published:** 2022-09-28

**Authors:** Conor Wall, Dylan Powell, Fraser Young, Aaron J. Zynda, Sam Stuart, Tracey Covassin, Alan Godfrey

**Affiliations:** 1 Department of Computer and Information Sciences, Northumbria University, Newcastle upon Tyne, United Kingdom; 2 Department of Kinesiology, Michigan State University, East Lansing, Michigan, United States of America; 3 Department of Sport, Exercise and Rehabilitation, Northumbria University, Newcastle upon Tyne, United Kingdom; Al-Balqa Applied University Prince Abdullah bin Ghazi Faculty of Information Technology, JORDAN

## Abstract

Mild traumatic brain injury (mTBI or concussion) is receiving increased attention due to the incidence in contact sports and limitations with subjective (pen and paper) diagnostic approaches. If an mTBI is undiagnosed and the athlete prematurely returns to play, it can result in serious short-term and/or long-term health complications. This demonstrates the importance of providing more reliable mTBI diagnostic tools to mitigate misdiagnosis. Accordingly, there is a need to develop reliable and efficient objective approaches with computationally robust diagnostic methods. Here in this pilot study, we propose the extraction of Mel Frequency Cepstral Coefficient (MFCC) features from audio recordings of speech that were collected from athletes engaging in rugby union who were diagnosed with an mTBI or not. These features were trained on our novel particle swarm optimised (PSO) bidirectional long short-term memory attention (Bi-LSTM-A) deep learning model. Little-to-no overfitting occurred during the training process, indicating strong reliability of the approach regarding the current test dataset classification results and future test data. Sensitivity and specificity to distinguish those with an mTBI were 94.7% and 86.2%, respectively, with an AUROC score of 0.904. This indicates a strong potential for the deep learning approach, with future improvements in classification results relying on more participant data and further innovations to the Bi-LSTM-A model to fully establish this approach as a pragmatic mTBI diagnostic tool.

## 1.0 Introduction

Globally, an estimated 69 million people suffer a traumatic brain injury (TBI) each year, with 81% of them being categorised as mild (mTBI, also termed a concussion) [1]. An mTBI can result in serious health implications, especially if misdiagnosed or poorly managed [2]. Research shows those who experience an mTBI, especially multiple occurrences in their early to midlife, have a higher risk of developing degenerative brain diseases later in life e.g., dementia [3].

Recognition and management of an mTBI in a non-invasive and timely manner is imperative to prevent more extreme symptoms and conditions or subsequent injury from occurring. Currently, the most common and practical method for diagnosing suspected mTBI in athletes is the subjective, pen and paper-based 5^th^ version of the Sport Concussion Assessment Tool (SCAT5) [4]. However, that method alone does not provide a conclusive diagnosis for mTBI and must be used in conjunction with other methods to provide a reliable diagnosis. In general, current reliable, objective methods of diagnosis are limited and none should be used in isolation due to the variance in mTBI severity and occurrence of symptoms, such as subjective reporting of e.g., headaches and nausea [5]. Ideally, a combination of tools would be used to increase the sensitivity and specificity of the diagnosis [6] but that multi-modal approach is impractical (cost, time, expertise), especially within low-resource settings, such as during community-based sports.

Accordingly, mTBI diagnosis is challenging and complex, even when using a combination of tools [7]. The lack of reliability and efficiency with traditional tools for diagnosis has led researchers to investigate and develop digital approaches, including nuanced computational methods [8]. For example, a mobile application was developed to perform a statistical analysis on various temporal and spectral speech-based acoustic features to find patterns indicative of mTBI [9]. In that study, features were extracted from speech-based data recordings consisting of tasks performed by those with an mTBI and control participants where certain combinations of the temporal and spectral features demonstrated great promise as potential biomarkers for identifying sustained mTBI from speech.

Speech analysis is complex, especially when considering real-world implications, such as the quality of the audio signal or accents. However, nuanced approaches with artificial intelligence (AI) such as deep learning may enable a robust analysis. For example, the use of a recurrent neural network (RNN) is one of the current leading methods for speech recognition and analysis. RNN architectures are particularly effective at dealing with sequential data, such as speech, due to their effectiveness in capturing important temporal patterns [10]. More recently, long short-term memory (LSTM) architectures (a format of RNN) have shown an improvement in advanced speech recognition when compared to more traditional RNN models [11].

Here we present a novel deep learning-based LSTM architecture to develop a methodology to recognise sports-related mTBI using audio classification in athletes. We hypothesise that this approach will be useful to identify athletes with an mTBI compared to those without.

The rest of the paper is organised as follows. Section 2 contains the related work to this study and the rationale behind the proposed methodology. Section 3 is an in-depth description of our proposed methodology, including participant information, data collection, feature extraction, optimisation, proposed model architecture, and model training process. Section 4 describes and illustrates the results of our investigation. Section 5 is the discussion of the results and the overall findings, while section 6 is the conclusion of the paper.

## 2.0 Related work and methodological rationale

### 2.1 SCAT5: Speech task

When administering the SCAT5, the participant undergoing the exam is instructed to read aloud an instruction paragraph to demonstrate any deficits in reading and speaking. The information recorded from this aspect of the test is included as part of the complete SCAT5 neurological screening component to identify receptive language disturbances [12]. Reading and speaking is manually assessed by a trained healthcare professional, such as a physical therapist. The paragraph read aloud by the participant is:

*The athlete should be given the symptom form and asked to read this instruction paragraph out loud then complete the symptom scale*. *For the baseline assessment*, *the athlete should rate his/her symptoms based on how he/she typically feels and for the post injury assessment the athlete should rate their symptoms at this point in time*.

A previous study [12] involving professional hockey players with an mTBI aimed to establish the accuracy and reliability of the SCAT5. Each component of the SCAT5 exam was assessed by a team physician or athletic trainer, including the instruction paragraph speech task (above). It was observed that only 1 of 140 mTBI participants had any difficulty reading the SCAT5 instruction paragraph. The study concluded that while some components of the SCAT5 provide a stronger indication of a sustained mTBI, the exam is generally subjective and should only be used to guide diagnosis. Additionally, the lack of sensitivity for the speech component can be derived from the manual approach and possible variation between assessors. Accordingly, manual delivery and interpretation for a speech-based task are limited and not conclusive enough to determine the potential effectiveness of the task. Alternatively, identifying speech biomarkers from audio recordings of the instruction paragraph using objective deep learning-based speech analysis could provide a stronger diagnostic approach.

### 2.2 Speech analysis: Neurological disorders

Analysis of speech data has demonstrated an increasing potential for diagnostic purposes concerning neurological disorders. Potential speech-based biomarkers, such as distorted vowels and hypernasality are often used [13]. Other speech features that have been analysed to determine if they are significant clinical biomarkers for neurological disorders include speech rate, articulation rate, silent/filled pause occurrence rate, and silent/filled pause length. Those speech features have shown to be extremely useful in differentiating between older adults with mild cognitive impairment and controls [14]. A Parkinson’s disease (PD) based study assessed the effectiveness of traditional and non-standard features to identify speech biomarkers [15]. That study introduced pitch period entropy (PPE) as a measure of dysphonia to differentiate people with PD from controls. The study found that the use of a combination of four measures and a kernel support vector machine (SVM), produced an optimal classification performance of all features utilised, with an overall classification result of 91.4%.

Robust identification of similar speech variations can provide a mechanism to identify those with mTBI [16]. In the referenced study, a total of seven speech tasks were included. Those tasks involved the reading aloud of specific sentences, such as the syllables ‘*pa*’, ‘*ka*’, and ‘*pa-ka-ta*’, and sustaining an ‘*aahhhh*’ sound for several seconds. From those tasks, a total of 38 different vocal features were investigated to assess if they indicate the existence of a brain injury, using a predictor value to illustrate the strength of each vocal feature. Specifically, that study used Hidden Markov Models [17] to find that the vocal feature with the most promising predictor value (0.372) with use of the average diadochokinetic (DDK) rate period. DDK-related tasks helped to assess the ability of the subject to produce syllables rapidly and in sequence i.e., if the participant had any issues with producing sounds, it could indicate the existence of an mTBI. However, all three DDK-related tasks produced a variety of results depending on the background of the participant, with their accent, age, sex, and potential neurodevelopmental conditions having an impact, regardless of having an mTBI. Furthermore, all three tasks were repeated three times at different speeds and were completed within 5–7 seconds for most participants. A more standardised and globally recognised approach with a longer sequence of speech being recorded and in a more consistent manner could provide more conclusive and consistent results.

### 2.3 Speech: Deep learning

Deep learning is an increasingly used AI-based technology to perform various classifications [18]. For example, it has been used to perform classification tasks, such as audio classification of respiratory diseases [19]. Of interest for this study is previous work examining an audio-based mTBI classification [20]. The referenced study employed the use of two machine learning models, one was a Gaussian Mixture Model (GMM), and the other was a Convolutional Neural Network (CNN) [21, 22]. Using participants categorised as having sustained an mTBI or not (i.e., controls), a range of speech (and gait) tasks were performed. From the speech data, a range of features were extracted from the recordings and subsequently trained on the GMM and CNN models. One of the speech features utilised during the study was Mel Frequency Cepstral Coefficient (MFCC), typically used for speech recognition and classification tasks and has shown robust proficiency for diagnostic medical purposes [23]. Results from Talkar et al. [20] show that the use of the speech features alone, the highest AUC score of 0.96 ± 0.05 was achieved using the CNN model, using features from three speech tasks (free speech, read speech, and ‘*pa-ta-ka*’). However, with 11 mTBI participants and 10 controls, the lack of variance in speech characteristics could lead to classification issues with future testing participants where their speech characteristics could differ from the original participants involved with the training model.

Using a more standardised and trusted approach, such as incorporating the speech tasks from the SCAT5 exam, would provide a better basis for model performance assessment and comparisons with other speech-based mTBI investigations.

### 2.4 Mel Frequency Cepstral Coefficient (MFCC)

**Fig 1:**
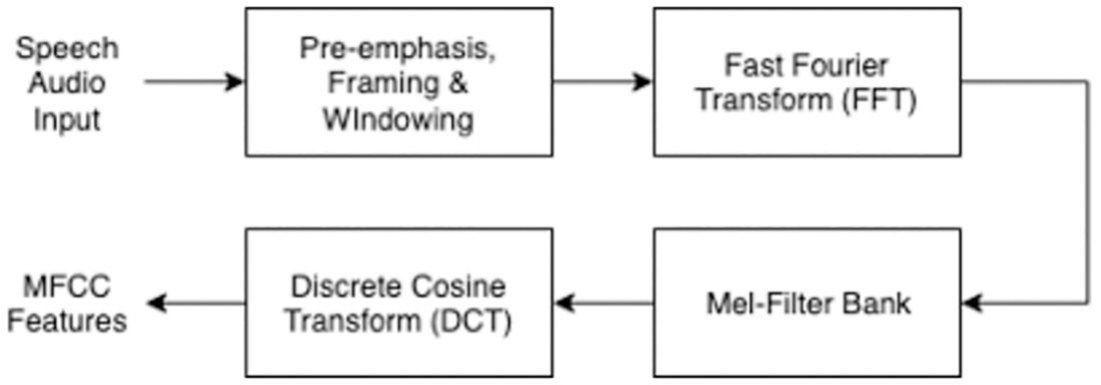
Four main steps of the MFCC feature extraction process.

MFCC features are commonly used for speech recognition and analysis tasks. The reasoning behind the widespread use of MFCC features for speech recognition is due to its (i) proficiency in error reduction, (ii) ability to extract robust features despite signal noise, and (iii) fundamental underpinnings based on the human perception of voice, which produces more realistic results [24, 25]. The process of extracting MFCC features is performed through four main computational steps, Fig 1 [26].

The pre-emphasis stage (Fig 1) is used to remove any noise from the frames. Subsequently, the overlapping of frames is performed during the framing stage to provide a smooth transition between each. Hanning windowing is then applied to cut off the end of each frame to provide additional smoothing between frames [27]. The fast Fourier transform (FFT) algorithm is subsequently performed on each frame to calculate the frequency spectrum using the Short-Time Fourier-Transform. On the Fourier transformed frames, the Mel-Filter bank is then applied to extract frequency bands using Eq 1 [26].

$$Mel\left(f\right)=C{\;log}_{10}\;\left(\;1+\frac{f}{f_{0}}\;\right)$$ (1)

Eq 1 is used by the Mel-Filter bank to approximate the human ear’s critical bandwidth and consists of numerous calculations. To calculate the constant (*C*), Eq 2 is used. The value of 1000, representing 1000Hz which equates to 1000 mels, is employed as well as the common logarithm (*log*_10_) and the corner frequency (*f*_0_) of between 600Hz and 1000Hz. To approximate the human ear’s critical bandwidth, a corner frequency of 700Hz is usually chosen.


$$C=\frac{1000}{{\;log}_{10}\left(1\;+\frac{1000}{700}\right)}$$ (2)

Following the calculation of the constant (*C*, 2595), all necessary values to perform Eq 1 are calculated, which can be seen in Eq 3. Here, the constant value of 2595 is multiplied by the common logarithm (*log*_10_), where a constant value of 1 is added to the result of the frequency in hertz (*f*) being divided by the corner frequency of 700Hz. The resulting value is the desired Mel-Filter bank output.


$$Mel\left(f\right)=2595{\;log}_{10}\;\left(1+\frac{f}{700}\right)$$ (3)

The final step is to compute the discrete cosine transform (DCT) of the Mel-Filter bank output, which outputs the cepstral coefficients in order of significance.

### 2.5 Improving deep learning performance

Deep learning has shown increasing promise for speech recognition [28]. One type of deep learning network that is commonly used for this purpose is the LSTM [29]. The use of an LSTM-based model with MFCC features for speech recognition has been demonstrated concerning Indonesian speech digit recognition [30]. Results from that study demonstrated a strong performance using the LSTM and MFCC methodology, with accuracy results of approx. 96%.

A technique used to improve the performance of RNN based models, such as LSTM, is the implementation of bidirectional RNN layers. A bidirectional RNN model contains two RNN layers of the same type, such as two LSTM layers. These two layers ensure that input characteristics may be processed in both forward and backwards directions, allowing better determination of the relationships between components in the input sequence [31].

Another technique explored to improve speech recognition performance with RNN technology is the implementation of an attention mechanism. The attention mechanism has the adaptive capacity to learn the relationship between each of the input features over numerous time steps to predict the current time step [32]. The effectiveness of the attention mechanism regarding the audio classification of two environmental sound datasets [33], ESC-50 and ESC-10 [34], using RNN technology was previously demonstrated by boosting accuracy performance by over 2% (for both datasets).

### 2.6 Synthesis of approaches

**Fig 2:**
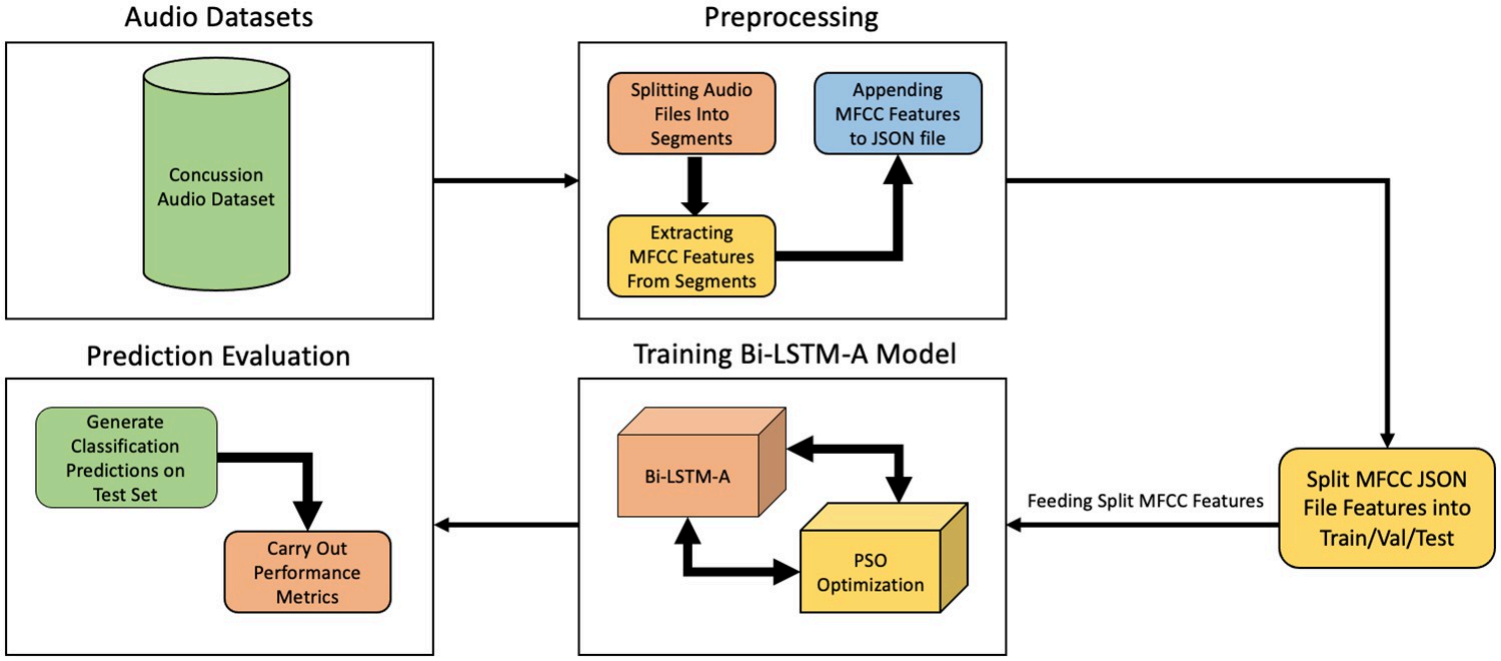
Bi-LMST-A model from audio dataset clockwise, displaying steps from the pre-processing phase to prediction evaluation phase.

Considering the literature reviewed here, several decisions regarding methodology were made for the purposes of this pilot study. It was concluded that the use of MFCC features for the feature extraction process would be most suitable due to its strong proficiency and performance in speech recognition tasks. Additionally, regarding the model of choice, we propose a bidirectional LSTM-based architecture that employs the use of an attention mechanism, or Bi-LSTM-A for short (Fig 2).

## 3.0 Methods

### 3.1 Participants

The work conducted is part of a larger and longitudinal study examining mTBI in rugby playing athletes. Ethical approval was granted by Northumbria University Research Ethics Committee (Ref: 23365). All participants gave informed written consent before providing data during the SCAT5 assessment with voice recording. For the purposes of this study, a total of 46 athletes were recruited from a university rugby team in the North East of England during the 2021 pre-season baseline testing period.

Of those athletes recruited, 7 were diagnosed with an mTBI. Those athletes were identified after sustaining a head injury, either self-reported or identified by a coach. Athletes were subsequently diagnosed with an mTBI by a medical doctor or physiotherapist through SCAT5 administration (within 8.2 ± 6.4 days of head impact). The inclusion and exclusion criteria for this study are from a multi-modal mTBI assessment protocol [35], Table 1.

**Table 1:** Participant inclusion and exclusion criteria.

*Inclusion Criteria*	*Exclusion Criteria*
≥18 years.	Be a pregnant female.
Have minimal cognitive impairment, defined as a score between 0 and 8 on the Short-Blessed test for cognitive function.	Be unable to abstain from medication/alcohol 24 hours in advance of testing
English as a first language or fluency.	Have history of peripheral vestibular pathology or eye movement deficits.
Those that have an mTBI/concussion during the season must have a diagnosis of mTBI from a healthcare professional (physiotherapist or medic) based upon standard criteria or identified head injury from their contact sport governing body.	Medical history of a neurological illness that could grossly affect balance or coordination (e.g.. stroke, greater than mild TBI, lower-extremity amputation, recent lower extremity or spine orthopaedic injury requiring a profile).

### 3.2 Data capture and pre-processing

All 46 participants were instructed to read aloud the same symptom evaluation instruction paragraph (*section 2*.*1*), as instructed in the SCAT5 exam, with all recordings using the Voice Analyst app [36] via an iPhone 11 with the sample rate of 44,100 Hz (44.1kHz). To eliminate any potential effects of other voices having a negative impact on the model training and classification performance, all recordings were manually clipped/truncated to contain the participant voice only.

### 3.3 Feature extraction

The feature extraction method of choice was MFCC and was achieved using the python package *Librosa* [37]. Prior to the feature extraction process, several variables were established which included (i) the sample rate, (ii) the number of cepstral coefficients, (iii) the length of the FFT window, and (iv) the hop length.

As the sample rate of each recording was 44.1kHz, the same value was used here. For the remainder of the variables, the default values provided in *Librosa* were used i.e., 13 for the number of cepstral coefficients, 2048 for the length of the FFT window, and 512 for the hop length.

The audio files were then segmented into several time frames, depending on the length of the speech clips (i.e., some participants read faster than others). Following the extraction of the MFCC features from the audio files, they were each appended onto a JSON file which is used as the input file for training the model architectures, Fig 2.

### 3.4 Optimisation

To find the optimal number of units for each layer, particle swarm optimization (PSO) was utilised. PSO is a widely used population-based heuristic optimization algorithm inspired by the social behaviour of bird flocking and fish schooling [38]. PSO has illustrated robustness to solve global optimization problems owing to its effective search strategies, model scalability, and robustness [39]. This enables the PSO algorithm to be suitable for optimizing hyperparameters used for training deep learning models, e.g., learning rate, momentum, and batch size [40]. PSO has shown that when compared to other optimisation methods, such as grid search, random search, Bayesian optimisation, and genetic algorithm, it can perform more optimally regarding hyperparameter tuning for various machine learning methods [41].

Each of the hyperparameters for our optimisation process had a set of pre-defined possibilities for values, of which the PSO algorithm then chose the most befitting for classification performance. Here, Algorithm 1 is a pseudocode representation of the PSO approach, which demonstrates the use of ‘particles’ in a ‘swarm’ to find the optimal values that have the best global fitness value for the set criteria, e.g., learning rate [42].

**Algorithm 1** PSO pseudo code

**Begin**

 **for** each particle in the swarm

  Initialize its position and velocity with random numbers

 **end for**

**do**

 **for** each particle in the swarm

  **if** current fitness value is better than current personal best fitness

  value, current fitness value becomes personal best fitness value

  **end if**

 **end for**

 For all particles in the swarm, choose the particle with the best fitness

 value as global best

 **for** each particle in swarm

  Update particle velocity

  Update particle position

 **end for**

**until stopping criteria is met**

**end begin**

### 3.5 Bi-LSTM-A model architecture

The model architecture designed for our approach is based on a synthesis of the research literature that has illustrated the proficiency of bidirectional RNN technology and attention mechanisms for audio and speech classification tasks. Table 2 details each of the layers implemented in the Bi-LSTM-A model, including the layer description and number of units chosen for each layer.

**Table 2:** Bi-LSTM-A model architecture.

*Layer*	*Layer Description*	*Units*
L1	Bidirectional LSTM	512
L2	LSTM	256
L3	Attention Mechanism	512
L4	Dense	128
L5	Dropout	0.6
L6	Dense	64
L7	Fully Connected Dense (*Softmax*)	2

In addition to the bidirectional LSTM layer, the LSTM layer, and the attention mechanism layer, a dropout layer was included to reduce the amount of overfitting that may occur during the training of deep learning models [43]. Early stopping was also implemented to determine the number of epochs during training and ultimately prevent any overfitting from occurring during the training process [44]. Three dense layers were also included, one being the fully connected layer. Dense layers are commonly used and are effective in RNN-based models, being deeply coupled with the layer that precedes it, which means that the neurons of the dense layer are connected to every neuron of the preceding layer [45].

### 3.6 Model training

Following the generation of the MFCC features using *Librosa*, the MFCC features were split at participant-level into train (60%), validation (20%), and test (20%) sets. Using the JSON file containing the MFCC features as the input features, the Bi-LSTM-A was trained with the PSO algorithm to optimise the model hyper-parameters. This includes the layer units, as well as the learning rate, momentum, and batch size. Table 3 showcases the optimized hyper-parameters found to be optimal for the training process.

**Table 3:** Bi-LSTM-A model optimised hyper-parameters.

*Hyper-Parameter*	*Value*
Learning rate	0.0001
Batch size	128
Epochs	89
Momentum	0.9

## 4.0 Results

**Fig 3:**
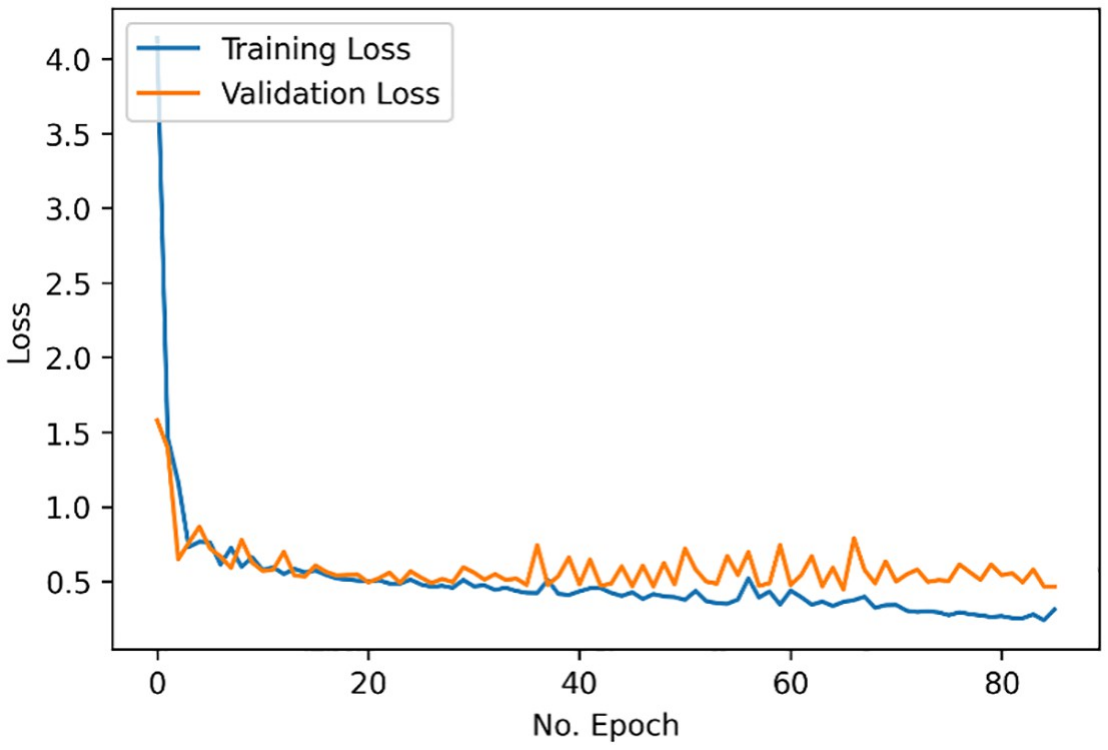
Bi-LSTM-A training and validation loss.

Following the feature extraction process and model training process, the classification results demonstrated below were computed. Little-to-no overfitting occurred during the training process, Fig 3. Consequently, a lack of overfitting ensures that the training process on observed data would allow the model to generalize well for classifying unseen data [46]. This indicates good reliability of the approach regarding the current test dataset classification results and future test data.

**Fig 4:**
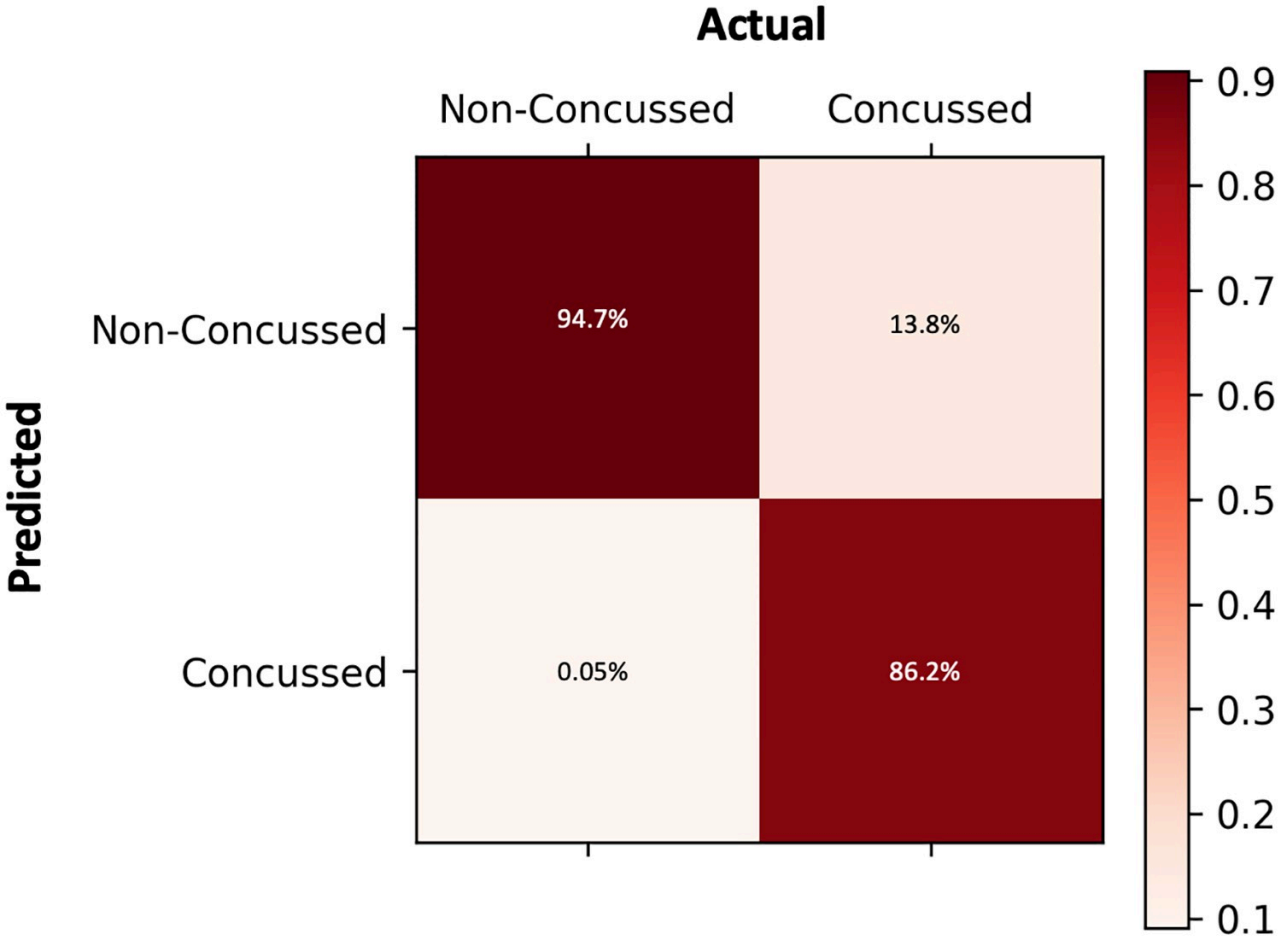
Confusion matrix of the Bi-LSTM-A model classification.

**Fig 5:**
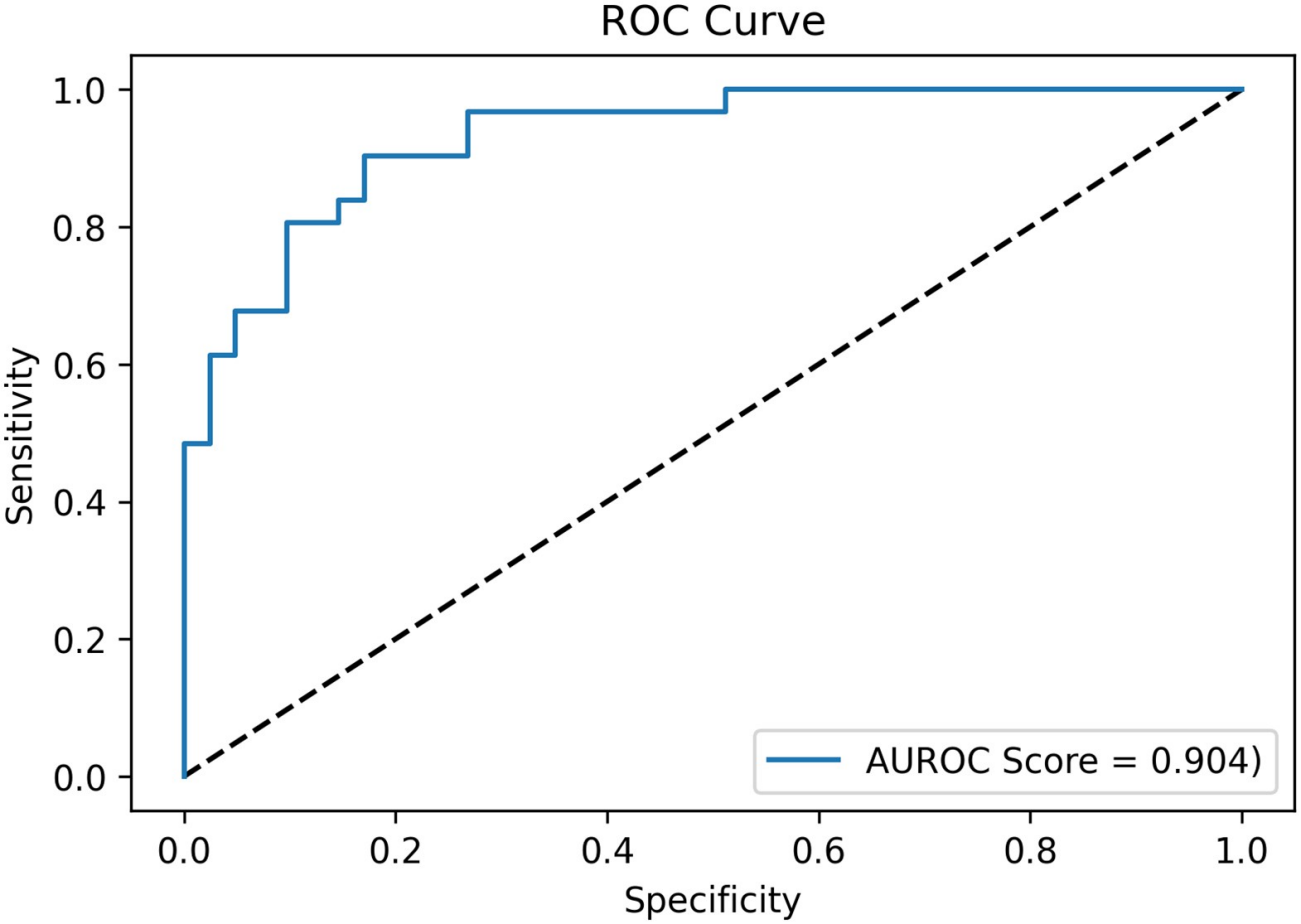
ROC curve of classification results.

From the proposed model architecture, there was an overall accuracy of 89.5% to identify those who had received an mTBI (from those with no mTBI). Fig 4 illustrates the classification results with a sensitivity of 94.7% and specificity of 86.2%. Fig 5 illustrates the classification performance with an ROC curve, as well as demonstrating the AUROC score of 0.904.

## 5.0 Discussion

Here we developed a deep learning-based model that provides an effective method (approx. 90%) that could be used to better inform mTBI/concussion diagnosis. Results from this pilot investigation suggest that the system could be an effective mTBI diagnostic tool with excellent sensitivity and specificity results, that compares positively to the current methods of mTBI diagnosis. The automated AI-based nature of the model means it could potentially be used in conjunction with other (digital) tools to provide a reliable and efficient assessment of mTBI.

Comparing our approach to a similar study [20], both sets of results show that there is strong promise regarding deep learning-based mTBI classification using speech data. Although the referenced study produced stronger results using a wider range of speech features, our results show the strong potential of using MFCC features and our Bi-LSTM-A model for mTBI diagnosis. One aspect of the study [20] that could be integrated into the methodology proposed here is the use of DDK-based tasks as an acoustic feature for improving classification performance. A combination of the SCAT5 instruction paragraph reading task and additional DDK-related tasks would provide a more rounded approach by assessing the various aspects of speech, such as speech rate, articulation rate, silent/filled pause occurrence rate, and silent/filled pause length, that have shown to be potential indicators of a sustained concussion.

Our preliminary results do indicate the existence of significant speech biomarkers that have been extracted using the MFCC technique from the athlete’s speech data. A more detailed and in-depth analysis into determining these specific biomarkers would be beneficial to further innovations made to the methodology regarding improving classification performance and computational efficiency. With research [16] showing that speech rate, articulation rate, silent/filled pause occurrence rate, and silent/filled pause length all being indicators of a neurological disorder, it is likely these acoustic features will be prevalent in a concussed athlete’s speech data.

To further analyse the performance of our model, we calculated the sensitivity and specificity values and then applied them to a confusion matrix, which can be seen in Fig 4. We also performed a receiver operating characteristic curve (ROC) analysis on the classification performance, as well as calculating the area under the receiver operating characteristic (AUROC) score, Fig 5. With the calculated AUROC score of 0.904, this demonstrates our model’s strong performance regarding this classification task, as any AUROC score above 0.9 is considered ‘outstanding’ [47]. Therefore, both metrics demonstrate our model’s strong performance at being able to use speech data to effectively distinguish participants with a mTBI compared to those without.

With the use of PSO optimization, a seamless transition could be made, while maintaining a high level of accuracy. This may have pragmatic use if scope was beyond sports-related mTBIs to diagnose mTBIs that were caused through other means such as older adult fallers. Although sports-related injuries account for a vast amount of observed mTBI injuries, the most common cause of mTBI injury is falling, with violence, bicycle accidents, and motor vehicle accidents also accounting for many mTBIs [48]. Our methodology could therefore potentially be used to provide a convenient and reliable diagnostic tool for anyone suspected of sustaining an mTBI.

When initially determining the structure of our model, we performed extensive investigations to discover which recent deep learning techniques displayed strong proficiency for speech classification tasks. This ultimately led to the design of our Bi-LSTM-A model. Although our investigation involved one model only, from the results demonstrated it can be concluded that the bidirectional RNN methodology and the implemented attention mechanism provided a strong classification performance. Additionally, it also showed that the use of MFCC features works well in conjunction with our model and that this is an approach that should be explored further.

### 5.1 Limitations

Though our approach showcases excellent results, certain limitations would hinder it for a real-life application. The first is the limited amount of data concerning athletes with a diagnosed mTBI. Furthermore, lack of variance in speech, i.e., accent, speaking style, speaker physiology, age, and emotions, within the dataset could potentially have a significant negative effect on speech recognition reliability [49]. With the sample being biased toward native English speakers from the North East of England, it, therefore, may not be representative of other locations, dialects, and accents. Consequently, gathering more participants from diverse backgrounds to produce more variance in data to train the model would be beneficial.

Another limitation is the recovery time of the tested athletes. As 45% of concussed athletes show clinical recovery within 14 days of injury, having athletes examined within a more acute time, i.e., within 48 hours of impact, rather than day 8 post-impact, would be more beneficial for analyzing aspects of speech that indicate the existence of an mTBI. Expanding the number of participants, as well as evaluating a more acute sample (i.e., within 48 hours), could therefore provide a greater number of speech biomarkers that signify the existence of an mTBI within a concussed athlete.

### 5.2 Future work

Future work will entail making further innovations to the Bi-LSTM-A model, including constant model optimization using the PSO algorithm whenever new participant data is added. In a similar vein, our approach could also be used with the development of an automated mTBI assessment system, using a smartphone to record and classify the voice data in real-time. Specifically, smartphone-based deployment of the trained Bi-LSTM-A model could provide a means of recording and classifying the recording during a break in or upon the conclusion of contact sports.

Here, a lightweight model containing only around 1 million parameters with an overall size of 11.6MB, was developed with the aim of providing an effective near real-time mTBI assessment within a smartphone. Our initial results suggest our proposed model could be effectively deployed on a smartphone. Although the scope of our investigation was predominantly concerned with the application of the SCAT5 exam within rugby, the nature of the methodology does not limit the assessment to one specific area and could be more flexible for use in other cohorts.

Ongoing work within the larger study is continuously collecting voice data to grow datasets, to refine the approaches presented here. Future work will test the suitability of the methodology presented here on newly acquired voice data, particularly in acute cases (e.g., mTBI <24hours). Moreover, work will also aim to test the methodology on independent datasets collected through the UK network of TBI researchers (https://tbi-research.uk/).

Low-cost digital deployments of this technology could be used to supplement routine mTBI protocols. Combining our deep learning-based voice analysis with additional data sources and protocols such as visual, cognitive, and motor assessments could ultimately improve the precision and proficiency of the mTBI diagnosis process [20]. This would also help significantly with the collection of valid and useful participant data. Equally, with the collection of more data to improve the AI approach, the model could be used as an mTBI tool in combination with other measures (e.g., gait/walking assessment).

## 6.0 Conclusion

Accurate diagnosis of mTBI is difficult due to the variance in symptoms and severity that can occur for different people. Current methods of mTBI diagnosis are not significantly reliable and are often required to be used collectively to increase the reliability of results. Ultimately, this is to prevent any brain injury from being undiagnosed, preventing further and more severe complications from occurring.

Here, we present an AI deep learning-based system, Bi-LSTM-A, that incorporates bidirectional, LSTM, and attention mechanism techniques, while being optimised by a PSO algorithm. Our system can distinguish those who have previously sustained an mTBI from those without by utilising MFCC features extracted from speech data using standardised SCAT5 text to an accuracy of approx. 90%. This provides a solid foundation for developing a highly effective and efficient mobile-based mTBI diagnosis tool.
